# Compact and programmable large-scale optical processor in free space

**DOI:** 10.1038/s41377-026-02236-2

**Published:** 2026-03-19

**Authors:** Maria Gorizia Ammendola, Nazanin Dehghan, Lukas Scarfe, Alessio D’Errico, Francesco Di Colandrea, Ebrahim Karimi, Filippo Cardano

**Affiliations:** 1 Scuola Superiore Meridionale, Via Mezzocannone, 4, 80138 Napoli, Italy; 2 Nexus for Quantum Technologies, University of Ottawa, K1N 5N6, Ottawa, ON Canada; 3 Dipartimento di Fisica “Ettore Pancini”, Università degli Studi di Napoli Federico II, Complesso Universitario di Monte Sant’Angelo, Via Cintia, 80126 Napoli, Italy; 4 National Research Council of Canada, 100 Sussex Drive, Ottawa, ON K1A 0R6 Canada; 5 Institute for Quantum Studies, Chapman University, Orange, CA 92866 USA

**Keywords:** Quantum optics, Optical materials and structures, Optical physics

## Abstract

Photonic circuits are central to classical and quantum information processing. While integrated technologies dominate, free-space architectures are emerging as attractive alternatives, offering broad bandwidth and direct manipulation of optical modes without confinement in waveguides. A key challenge for scalability lies in circuit depth, as the number of layers manipulating the optical field typically grows with the system size. Here, we introduce a programmable free-space photonic platform that implements translation-invariant, high-dimensional unitary transformations using only three layers. Information is encoded in structured light modes defined by circular polarization and quantized transverse momenta, and processed with spatial light modulators interleaved with half-wave plates. We implement unitaries that are equivalent to quantum walks over up to 30 time steps, in one- and two-dimensional lattices, distributing a single input mode across more than 7,000 outputs, where conventional approaches would require tens or hundreds of layers. The platform supports diverse quantum walk dynamics, including disorder, synthetic gauge fields, and topological effects, previously explored only in separate experiments. Using coincidence detection with a time-tagging camera, we show compatibility with quantum optics protocols and provide examples of quantum walks of heralded single photons. These results contribute to establishing free-space optical processors as promising resources for high-dimensional quantum simulation and scalable optical information processing.

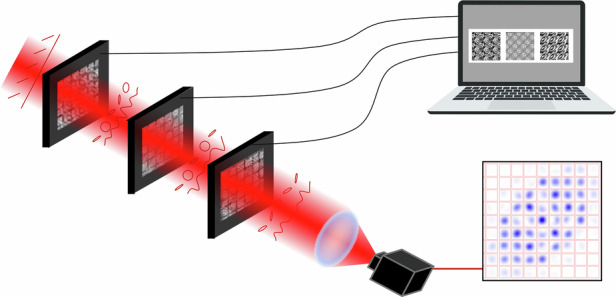

## Abstract (web summary)

A programmable three-layer photonic platform implements large-scale unitary transformations, distributing the input field into thousands of output modes for scalable photonic information processing in free space.

## Introduction

Programmable photonic platforms are versatile tools for classical and quantum technologies, enabling applications in communication^1^, information processing^2^, and simulation^3^. They are considered essential building blocks for all-optical quantum computers^4^ and photonic neural networks^5^, thanks to their ability to manipulate spatial, temporal, spectral, and polarization degrees of freedom. Integrated circuits exploit waveguide arrays with beam splitters and phase shifters to couple spatial modes^6,7^, while temporal modes are controlled with integrated optics^8^, fiber loops^9^, or birefringent materials for ultrafast operations^10^. Alternatively, multimode fibers combined with spatial light modulators (SLMs) give access to large Hilbert spaces of transverse modes, supporting high-dimensional entanglement^11^, quantum walks (QWs)^12^, and reconfigurable quantum networks^13,14^.

Free-space optical processors offer a compelling alternative to integrated solutions, providing flexible access to many co-propagating structured modes. By combining programmable phase masks with free-space propagation^15^, they have attracted interest for photonic neural networks^16–18^ and high-dimensional quantum photonics^19^. Examples of achievable tasks are mode sorting, high-dimensional quantum gates, entanglement certification, and photonic quantum computing^20–25^. However, their scalability is limited by circuit depth and optical losses. Here, we address this challenge by introducing a reconfigurable free-space platform based on liquid-crystal (LC) SLMs and an analytical inverse-design method, enabling exact unitary transformations with only three patterned layers.

Diffractive elements implementing space-variant polarization manipulation^26^ have further expanded the toolbox for free-space processors, with applications including optical computing^27^ and holography^28–30^. A fully vectorial multi-plane light converter (MPLC) system has been demonstrated recently^31^, implemented via a sequence of dielectric metasurfaces, interleaved with free-space propagation, to realize mode conversion between vectorial states of light. However, in these multilayer architectures, the platform depth scales with the number of modes, making low-loss, accurate, and programmable implementations a central challenge^19^. To mitigate this, recent studies have focused on resource-efficient few-layer platforms. Single-layer dielectric metasurfaces have implemented generalized beam splitters^32^, C-NOT gates^33^, and multiphoton state characterization^34^. Complementarily, by using multi-layer patterned static LC devices, an efficient compression scheme for implementing large-scale translation-invariant unitaries in one- and two-dimensional spatial configurations using only three patterned layers has been demonstrated^35,36^.

Here, we demonstrate that a similar compression technique can be implemented via commercially available LC-based SLMs, thereby realizing reconfigurable photonic circuits in free space. While the previous setups with static LC devices relied on the possibility of patterning the LC molecular director in the transverse plane^35,36^, the new scheme achieves dynamic programmability via remote control of the local out-of-plane orientation of LC molecules. A remarkable result is that the method retains the possibility of extracting analytical solutions for the patterned layers, as in the previous schemes, which avoids the use of iterative optimization approaches, typically adopted in MPLCs^20,21,25^ or propagation through complex media^11,14^. The platform thus allows, in principle, exact realization of arbitrary translation-invariant unitaries with only three reconfigurable, patterned devices.

Although SLMs are usually employed as phase-only elements on scalar fields^37^, their use for space-dependent polarization transformations has been proposed with cascaded configurations^38–40^ and partially demonstrated with a single device^38^. The platform presented here provides full control over both the spatial and vectorial degrees of freedom of light, without the need to select a specific polarization component or diffraction order.

While this work was in preparation, an experimental realization of such transformations for structured light was reported in a related setup^41^. In combination with our results, these advances establish the first complete reconfigurable platform for arbitrary, space-dependent polarization transformations. Our work also represents the first implementation of such an architecture as a photonic circuit on spin-orbit modes, within the class of unitary matrices featuring discrete translation invariance. This establishes a novel paradigm for optical information processing and quantum simulation with structured light in free space.

We validate the platform by implementing over 300 distinct mode-coupling unitaries in the form of coined QWs^42^, while maintaining fixed and shallow circuit depth. We further demonstrate its suitability for quantum optics by integrating event-based, single-photon-sensitive cameras that simultaneously resolve transverse positions and photon arrival times with nanosecond resolution^43^. This extends previous studies with static LC devices, which typically involved fewer modes and classical laser light. Together, these results represent a significant step toward stable, efficient, and scalable free-space photonic circuits.

## Results

### Coupling transverse momentum modes via SLMs

Our circuit processes optical modes having the following expression:

1 $$| {m}_{x},{m}_{y},j\rangle =A(x,y,z){e}^{i{k}_{z}z}{e}^{i({m}_{x}x+{m}_{y}y)\Delta {k}_{\perp }}| j\rangle$$

where (*x*, *y*) are the coordinates in the transverse plane, with photons assumed to propagate along *z*. Here, *A*(*x*, *y*, *z*) is a Gaussian envelope, *k*_*z*_ is the wavevector *z*-component, Δ*k*_⊥_ is a unit of transverse momentum, $| j\rangle$ is a polarization state, which can be left-handed $| L\rangle ={(1,0)}^{T}$ or right-handed $| R\rangle ={(0,1)}^{T}$, and (*m*_*x*_, *m*_*y*_) are integer numbers.

**Fig. 1:**
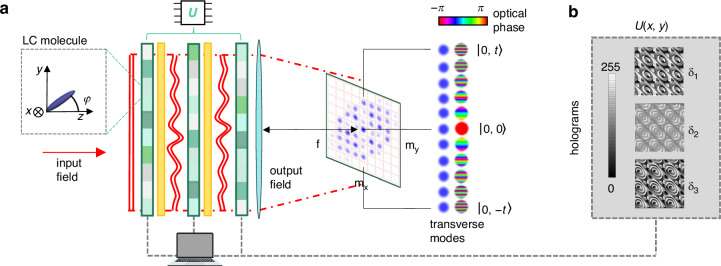
Programmable photonic circuit based on spatial light modulators. **a** Conceptual overview of the platform. The input field $| 0,0,j\rangle$ propagates through three SLMs, alternating with two HWPs (yellow), implementing the optical transformation corresponding to the target circuit. The output field modal distribution is revealed in the focal plane of a lens, where the optical intensity pattern is recorded on a camera. Photonic modes at the circuit input and output carry (*m*_*x*_, *m*_*y*_) units of transverse momentum Δ*k*_⊥_ along the *x* and *y* directions, respectively, resulting in linear phase gradients across the transverse *xy* plane. For simplicity, a 1D cut along *m*_*x*_ = 0 is shown at the output plane. Optical polarization provides an additional degree of freedom, effectively doubling the dimensionality of the mode space. The inset shows the local orientation of the LC molecules within the SLM. The local birefringent retardation *δ*(*x*, *y*) depends on the local out-of-plane orientation *φ*. **b** A dedicated software computes the holograms for each SLM and displays them on three SLMs, enabling real-time reprogramming of the circuit for specific target unitaries

A conceptual scheme of the setup is sketched in Fig. 1a. Starting from a single spatial mode, e.g. $| 0,0\rangle$, the application of a unitary map *U*, invariant under discrete translations in the Hilbert space spanned by $| {m}_{x},{m}_{y}\rangle$, results in a superposition of output modes:

2 $$U| 0,0,j\rangle =\mathop{\sum }\limits_{{m}_{x}^{{\prime} },{m}_{y}^{{\prime} }=-t}^{t}\mathop{\sum }\limits_{h\in \{L,R\}}{c}_{{m}_{x}^{{\prime} },{m}_{y}^{{\prime} },h}| {m}_{x}^{{\prime} },{m}_{y}^{{\prime} },h\rangle$$

where *t* sets the range of transverse modes coupled by *U*. Consequently, when considering a localized input state, the total number of addressable transverse modes via *U* is *d*^2^ = (2*t*+1)^2^. Measuring the optical field in the focal plane of a lens, where the modes defined in Eq. (1) are spatially resolved, enables rapid and direct retrieval of the field distribution across the circuit’s optical modes, without requiring additional mode projection or scanning. Importantly, negligible overlap between neighboring modes is guaranteed as long as *w*_0_≥Λ^44^, where *w*_0_ is the Gaussian beam waist.

Our photonic circuit implements the target unitary *U* as a coined QW, a widely used model for discrete-time quantum dynamics on a lattice with an internal degree of freedom, referred to as the coin. The QW describes a sequence of unitary operations that combine coin rotations with coin-dependent translations across lattice sites (more details below). In our implementation, lattice sites correspond to spatial modes $| {m}_{x},{m}_{y}\rangle$ and coin states to circular polarizations $| j=L,R\rangle$^44^. This encoding enables a compact implementation of translation-invariant unitary maps via space-dependent polarization transformations^35,36^ (see Methods for details). In this framework, the target unitary operator can also be modeled as a position-dependent polarization transformation acting on the structured light field, and takes the form:

3 $$U=\iint {\mathrm{d}}x\,{\mathrm{d}}y\,\,{\mathcal{U}}(x,y)\otimes | x,y\rangle \langle x,y|$$

where ${\mathcal{U}}(x,y)$ is an arbitrary SU(2) matrix, defined by three real parameters. The translation invariance of *U* implies that ${\mathcal{U}}(x,y)$ is periodic with period Λ = 2*π*/Δ*k*_⊥_, and contains spatial frequency components corresponding to quantized values of the transverse momentum unit ± Δ*k*_⊥_^35^. Put simply, the effect of this polarization transformation can be viewed as polarization-controlled diffraction, imparting momentum kicks to photons in discrete steps of Δ*k*_⊥_, which matches the spacing between neighboring modes in momentum space (see Eq. (1)).

Previous studies have shown that ${\mathcal{U}}(x,y)$ can be realized via a minimal set of three waveplates with patterned optic-axis orientations, implementable using liquid-crystal metasurfaces (LCMSs)^35,36^. Each metasurface acts as a standard waveplate with a spatially varying optic axis, *θ*(*x*, *y*), given by the in-plane orientation of LC molecules with respect to the *x* axis, and uniform yet tunable birefringence *δ*. By applying an electric field to the plate, LC molecules are tilted out-of-plane toward the propagation direction, which allows for controlling the phase difference between the ordinary and extraordinary components, as shown in the inset of Fig. 1a. The metasurfaces’ patterns *θ*_*i*_(*x*, *y*) (*i* = {1, 2, 3}) realizing the transformation of Eq. (3) are then found by imposing

4 $${\mathcal{U}}(x,y)={Q}_{{\theta }_{3}(x,y)}(\pi /2){Q}_{{\theta }_{2}(x,y)}(\pi ){Q}_{{\theta }_{1}(x,y)}(\pi /2)$$

where

5 $${Q}_{\theta }(\delta )=\left(\begin{array}{cc}\cos (\frac{\delta }{2}) & i\sin (\frac{\delta }{2}){e}^{-2i\theta }\\ i\sin (\frac{\delta }{2}){e}^{2i\theta } & \cos (\frac{\delta }{2})\end{array}\right)$$

is the standard waveplate Jones matrix in the circular polarization basis.

However, such a circuit is a static machine capable of implementing only the target operation. To achieve reconfigurability, we coherently replaced LCMSs with SLMs. Essentially, an SLM consists of a pixelated array of LC-filled cells, each of which can be controlled independently via software^45,46^. In the circular polarization basis, its Jones matrix can be written as

6 $${S}_{\theta }(\delta (x,y))={e}^{i\frac{\delta (x,y)}{2}}{Q}_{\theta }(\delta (x,y))$$

where, opposite to LCMSs, *δ* can be locally controlled by applying a different electric voltage at each pixel, while the in-plane orientation *θ* is uniform. In the following, we assume *θ* = 0. SLMs are traditionally employed as digital phase retarders inducing a phase shift *δ*(*x*, *y*) to the incoming $| H\rangle$-polarized beam, where $| H\rangle =(| L\rangle +| R\rangle )/\sqrt{2}$ is the horizontal polarization, that is parallel to the SLM optic axis. The voltage, and hence the phase shift, can be controlled using grayscale images, referred to as holograms, usually with an 8-bit encoding, which allows for 255 phase levels. Here, we exploit the possibility of programming the local birefringence parameter of SLMs in order to use them as polarization-controlling devices, effectively acting as waveplates with space-dependent optical retardation. At each transverse position, the target unitary ${\mathcal{U}}(x,y)$ can be implemented via three SLMs, locally programmed to satisfy the equation

7 $${\mathcal{U}}(x,y)={e}^{-i\frac{{\delta }_{4}}{2}}{S}_{0}({\delta }_{3}){H}_{2}{S}_{0}({\delta }_{2}){H}_{1}{S}_{0}({\delta }_{1})$$

where *δ*_4_ = *δ*_1_ + *δ*_2_ + *δ*_3_, *H*_1_ = *Q*_*π*/8_(*π*) and *H*_2_ = *Q*_−*π*/8_(*π*) are two half-wave plates (HWPs), and we omitted the spatial dependence of each *δ*_*i*_ (*i* = {1, 2, 3, 4}) on (*x*, *y*) for ease of notation. The intermediate HWPs are necessary to prevent the trivial effect of three cascaded SLMs, which would result in a mere phase transformation on the $| H\rangle$ polarization component.

The global phase − *δ*_4_/2 in Eq. (7) is required to cancel the extra space-dependent global phases accumulated after each SLM bounce (see Eq. (6)). Without compensation, the three-SLM transformation would be a complex U(2) transformation rather than an SU(2) one, thus deviating from Eq. (3). A simple way to cancel this phase could be that of leveraging a fourth SLM at the input of the circuit, which adds the phase − *δ*_4_(*x*, *y*)/2 to an incoming $| H\rangle$-polarized beam. The input coin-polarization state could then be rotated and adjusted according to the target simulation. This approach would require an additional layer in our circuit. An alternative solution, which we adopt here, allows us to keep the circuit depth at a minimum of three layers.

In our experiment, for practical reasons, we always set $| H\rangle$ at the input of the first SLM, and superimpose the cancellation phase mask to the hologram *δ*_1_(*x*, *y*) precomputed from Eq. (7):

8 $${S}_{0}({\delta }_{1})\to {S}_{0}({\widetilde{\delta }}_{1})$$

with ${\widetilde{\delta }}_{1}={\delta }_{1}-{\delta }_{4}/2$. This setting, however, does not prevent us from also simulating the output of a different polarization input, say $U| \phi \rangle$, with $| \phi \rangle =\Omega | H\rangle$. In this case, we implement the rotated operation

9 $${{\mathcal{U}}}^{{\prime} }(x,y)| H \rangle ={S}_{0}({\delta }_{3}){H}_{2}{S}_{0}({\delta }_{2}){H}_{1}{S}_{0}({\mathop{\delta }\limits^{ \sim }}_{1})| H\rangle$$

where ${{\mathcal{U}}}^{{\prime} }={\mathcal{U}}\Omega$, with the physical input of the circuit always being $| H\rangle$.

The analytical solutions of Eq. (7), yielding the set of holograms for a given unitary *U*, are provided in Methods. Via software, these solutions are uploaded and displayed on the SLMs (see Fig. 1b). Remarkably, the availability of closed-form, analytical expressions provides a significant computational advantage, as it enables the direct extraction of the holograms without the need for optimization routines or iterative strategies.

To eliminate the undesired effect of free-space propagation between consecutive SLMs, we interpose two imaging 4-*f* systems between them. A complete description of the experimental setup is also provided in Methods. Ideally, in case SLMs could be placed very close to each other (not possible with currently available, bulky LC-SLMs), and with the fast axis of the central one rotated by 22.5°, there would be no need for lenses and HWPs.

### Experimental results

#### Quantum walks in 1D and 2D

We realize multiple steps of different QW processes across 1D and 2D lattices, also observing the effects of time-dependent step operators on the spreading of the wavefunction. Additionally, we show how the platform can be engineered to simulate either localized input states or wavepackets, without making any changes in the experimental setup, thus allowing us to probe geometrical and topological features in chiral-symmetric processes. Finally, we test the platform in the single-photon regime, validating its suitability for quantum experiments. In all experiments, the relative alignment between the phase holograms is fixed, and the local birefringence of each SLM remains constant over the typical experimental timescale. This ensures a high degree of stability and reliability, such that the error bars are typically smaller than the datapoints.

In our encoding, the walker lattice is spanned by the transverse modes introduced in Eq. (1), while circular polarization states encode a two-level coin. Each run of the experiment corresponds to a fixed time step *t* of the QW dynamics. In this implementation, the SLMs display the three holograms *δ*_*i*_(*x*, *y*) corresponding to the unitary $U(t)={U}_{0}^{t}$, where *U*_0_ is the single-step QW evolution operator and *t* is the number of time steps. Specifically, we focus on the QWs introduced in ref. ^47^ and ref. ^44^ for the 1D and 2D configuration, respectively, where the single-step evolution operators are

10 $$\begin{array}{rcl}{U}_{1}({\alpha }_{1}) & = & M{T}_{x}({\alpha }_{1})W{M}^{\dagger },\\ {U}_{2}({\alpha }_{2}) & = & {T}_{y}({\alpha }_{2}){T}_{x}({\alpha }_{2})W\end{array}$$

Here, *W* and *M* are coin rotation operators, reading

11 $$W=\frac{1}{\sqrt{2}}\left(\begin{array}{cc}1 & i\\ i & 1\end{array}\right)$$

and

12 $$M=\left(\begin{array}{cc}\cos (\pi /8) & i\sin (\pi /8)\\ i\sin (\pi /8) & \cos (\pi /8)\end{array}\right)$$

The matrix *M*, not appearing in the original work^47^, is here introduced as it simplifies the measurements of topological invariants that we will present later. The operator

13 $$\begin{array}{rcl} & & {T}_{x}(\alpha )=\cos (\alpha /2){{\mathbb{I}}}_{c}\otimes {{\mathbb{I}}}_{w}+i\sin (\alpha /2)\\ & & \mathop{\sum }\limits_{{m}_{x}}| R\rangle \langle L| \otimes {\widehat{t}}_{x}^{\dagger }+| L\rangle \langle R| \otimes {\widehat{t}}_{x}\end{array}$$

is the coin-dependent translation operator along *m*_*x*_, where ${\widehat{t}}_{x}| {m}_{x}\rangle =| {m}_{x}-1\rangle$, and ${{\mathbb{I}}}_{c}\otimes {{\mathbb{I}}}_{w}$ is the identity operator acting on the coin and walker space. A similar expression holds for *T*_*y*_(*α*). From the expression of the translation operator in Eq. (13), we observe that the parameter *α* tunes the hopping amplitudes between neighboring sites. In the experiment, we set *α*_1_ = *π* and *α*_2_ = *π*/2.

**Fig. 2:**
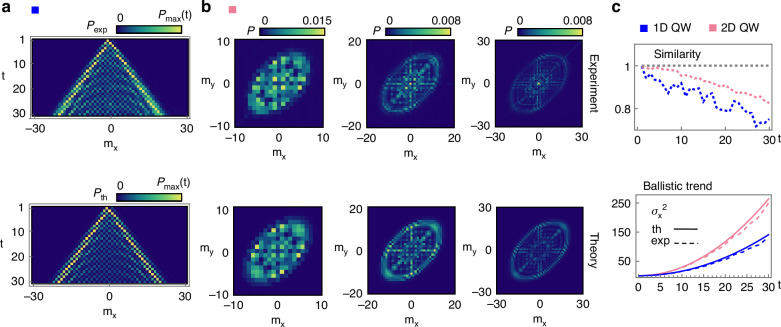
One- and two-dimensional QWs implemented via three SLMs. **a** Experimental ${P}_{\exp }$ (top) and theoretical *P*_th_ (bottom) probability distributions for 30 time steps of the 1D QW protocol *U*_1_, with input state $| L\rangle$. At each time step, the probability distributions are normalized with respect to the maximum probability at that time. **b** Experimental (top) and theoretical (bottom) probability distributions *P* for 30 time steps of the 2D QW protocol *U*_2_, with input state $| H\rangle$. Representative distributions are shown for time steps that are multiples of 10. The full time-resolved 2D dynamics is available in the Supplementary Material. **c** Similarity (top) and corresponding trend of the variance of the output distribution (bottom) for the 1D (blue) and the 2D (pink) QWs. The experimental datapoints (dots) correctly reproduce the expected ballistic behavior (solid lines) extracted numerically

The input is a localized walker state, obtained by preparing a Gaussian beam with beam waist *w*_0_/Λ ≥ 1^44^. Specifically, we set *w*_0_ ≃ Λ = 4 mm. The source is an 810 nm diode laser coupled to a single-mode fiber for spatial filtering (see Methods). At the output of the circuit, the QW distribution can be resolved in the focal plane of a lens placed after the last SLM. Each light spot is associated with a walker site, with probability given by the integrated light intensity within that spot, normalized with respect to the total light power. The procedure to simultaneously control the three SLMs and extract the output probability distribution in real-time is outlined in Methods. The agreement between the theoretical predictions and the experimental observations is quantified in terms of the similarity

14 $$S={\left(\mathop{\sum }\limits_{{m}_{x},{m}_{y}}\sqrt{{P}_{\exp }({m}_{x},{m}_{y}){P}_{{\mathrm{th}}}({m}_{x},{m}_{y})}\right)}^{2}$$

where ${P}_{\exp }$ and *P*_th_ are the normalized experimental and theoretical probability distributions, respectively. Figure 2a, b shows the experimental distributions obtained for the 1D and 2D protocols described above, for simulated input states $| 0,L\rangle$ and $| 0,0,H\rangle$, respectively, compared with theoretical predictions. We report the simulations of up to 30 steps in both realizations. In the 2D case, this corresponds to a QW activating up to $2* {(2* 30+1)}^{2}\,\simeq \,7400$ modes, where the factor 2 accounts for the polarization degree of freedom. Figure 2a shows the complete step-by-step 1D dynamics, while only multiples of 10 steps are reported in Fig. 2b for the 2D case. The full time-resolved 2D dynamics can be found in the Supplementary Material. The specific orientation of the walker distribution reflects the structure of the *U*_2_ protocol, missing a coin rotation between consecutive translations along the *m*_*x*_ and *m*_*y*_ directions. When adding such an additional operation, the walker symmetrically spreads across the entire lattice^48^.

This step-by-step analysis showcases the advantage provided by reconfigurability compared to the previous LCMS-based static platform^35,36^, which would have required fabricating a different set of three plates for each target simulation. Moreover, in contrast to typical optical setups for quantum simulations, our scheme enables the simulation of increasingly large numbers of steps without adding single-step optical elements, but simply updating the SLMs’ holograms. Accordingly, the circuit efficiency does not decrease with the number of steps, since the number of optical components stays constant, with an average recorded total efficiency of (52 ± 4)%, measured from the input to the output of the platform. The dominant contribution to the losses is associated with the third SLM, which exhibits a lower measured reflection compared to the other devices. The experimental characterization of reflection losses can be found in the Supplementary Material. The decrease in similarity values with higher step numbers, shown in the top panel in Fig. 2c, is mainly ascribed to the reduced resolution and larger pixel size of the last SLM used in this experiment compared to the first two, leading to aliasing effects. Possible errors in the phase calibration of the SLMs can also degrade the circuit performance (see Methods).

A key feature of the QW is that its probability distribution spreads faster than the classical random walk. In particular, the variance of the distribution scales quadratically with the number of steps, *σ*^2^ ∝ *t*^2^, which makes the QW a ballistic process. The bottom panel in Fig. 2c shows the variances extracted experimentally at each step in the two realizations. In the 2D case, the variance along the *x* direction is chosen for reference.

#### Time-dependent dynamics

In the previous section, we assumed the single-step operator to be identical at each step, though this condition can be relaxed to obtain more general transformations associated with time-dependent QWs. These processes can be employed to simulate temporal disorder and external fields, as we show in the following.

**Fig. 3:**
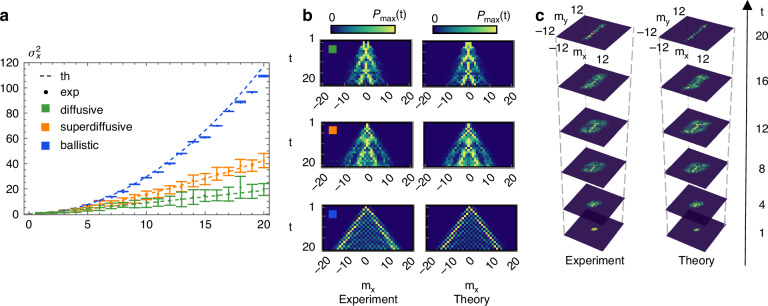
Dynamical disorder and electric QWs. **a** Theoretical (dashed) and experimental (solid) trend of the variance of the walker distributions in diffusive (green), superdiffusive (orange), and ballistic (blue) regimes. The experimental values of the variance are averaged over 5 disorder realizations. **b** Experimental (left) and theoretical (right) output probability distributions for a 1D QW with temporal disorder in one representative diffusive (top), superdiffusive (middle), and ballistic (bottom) configuration. At each time step, the probability distributions are normalized with respect to the maximum probability at that time. For each regime, the remaining 4 disorder configurations can be found in the Supplementary Material. **c** Experimental (left) and theoretical (right) probability distributions showing directional refocusing induced by a constant electric field in a 2D QW (*U*_2_). Representative distributions are shown for time steps that are multiples of 4. The full-time-resolved 2D dynamics is available in the Supplementary Material

By introducing temporal disorder in a QW, it is possible to modify its variance behavior, *σ*^2^(*t*) ∝ *t*^*β*^^42^. By varying the disorder strength, we experimentally investigated the transition from ballistic (*β* = 2) to diffusive (*β* = 1), which is a clear transition from a quantum-to-classical stochastic process, also accessing the intermediate superdiffusive regime (1 < *β* < 2). Disorder is introduced by simulating a time-dependent translation operator:

15 $$U(t)=\mathop{\prod }\limits_{n=1}^{t}{U}_{1}(n)=\mathop{\prod }\limits_{n=1}^{t}{T}_{x}({\alpha }_{1}(n))W$$

with *α*_1_(*n*) randomly extracted from the range $[{\overline{\alpha }}_{1}-\Delta \pi ,{\overline{\alpha }}_{1}+\Delta \pi ]$ at each step, where ${\overline{\alpha }}_{1}=\pi /2$ and Δ gives the degree of disorder^49^. Experimental data in Fig. 3a are in excellent agreement with the theoretical prediction (dashed lines) for the three chosen disorder strengths. The input is a localized $| 0,H\rangle$ state. Each datapoint is obtained as the average over 5 different realizations within each disorder regime, with error bars given by the corresponding standard deviations.

Table 1 reports the chosen values of Δ and the values of *β* obtained by fitting the experimental data (${\beta }_{\exp }$), compared to the values obtained from numerical simulations (*β*_th_) of the same disordered evolutions. For each regime, we perform a weighted fit of the datapoints ${\sigma }^{2}(n)={c}_{1}+{c}_{2}{n}^{{\beta }_{\exp }}$, with *n* the step number. The fit is used to estimate ${\beta }_{\exp }$ and its uncertainty.

**Table 1:** Expected theoretical (*β*_th_) and experimental (*β*_exp_) values of the exponent *β* for increasing disorder strengths (Δ), inducing the transition from ballistic to superdiffusive to diffusive spreading

	Δ	*β*_th_	*β*_exp_
ballistic	0%	1.99	1.92 ± 0.05
superdiffusive	37.5%	1.5	1.4 ± 0.1
diffusive	87.5%	1.2	1.2 ± 0.3

We perform a weighted fit of the datapoints ${\sigma }^{2}(n)={c}_{1}+{c}_{2}{n}^{{\beta }_{\exp }}$, with *n* the step number. The fit is used to estimate ${\beta }_{\exp }$ and its uncertainty

The programmability of our platform enables a time-resolved investigation of this transition encompassing up to 30 steps, surpassing previous experimental demonstrations^49^. Figure 3b shows experimental distributions for one value of Δ for each disorder strength. All the remaining data can be found in the Supplementary Material.

When an external force is applied to the walker, the QW can be used to simulate the effect of an electric field on a charged particle. Here, we reveal this effect in a 2D electric walk. As shown in previous works^44,50^, the application of an external constant force *F*_*x*_ is equivalent to a transverse displacement of the single-step evolution operator according to:

16 $${\mathcal{U}}(x,y,t)=\mathop{\prod }\limits_{n=0}^{t}{{\mathcal{U}}}_{0}(x+\Delta x(n),y)$$

where Δ*x*(*n*) = *nF*_*x*_Λ/(2*π*). In our experiment, we set *U*_0_ = *U*_2_(*π*/2) (see Eq. (10)). The energy bands resulting from this protocol exhibit an energy gap *E*_*g*_ ≈ 1^44^, which is sufficiently larger than the applied force *F*_*x*_ = *π*/10, thereby ensuring the validity of the adiabatic approximation. Under this approximation, an input state localized in the position space $| {m}_{x},{m}_{y}\rangle$ refocuses in the direction of the applied force with the typical period of Bloch oscillations *T* = 2*π*/*F*_*x*_ = 20 time steps^51^. This effect is well captured by our experimental simulation of 20 steps of the evolution of a localized $| 0,0,H\rangle$ input, as shown in Fig. 3c, where only multiples of 4 steps are reported. The full time-resolved dynamics can be found in the Supplementary Material.

#### Topological quantum walks

QWs provide a paradigmatic example of a periodically driven (Floquet) system that can be engineered to host all topological phases of single-particle systems^52^. These are characterized by quantized global features, known as topological invariants, underlying remarkable physical phenomena, such as robust edge states and quantized transport^53^. For instance, the 1D QW protocol *U*_1_ defined in Eq. (10) exhibits chiral symmetry, characterized by the existence of a unitary operator *Γ* that pairs states with opposite energy. This symmetry implies the quantization of the Zak phase: *φ*_Z_ = *νπ*/2, where *ν* is an integer, called the winding number, playing the role of a symmetry-protected topological invariant^52,54,55^. By choosing the single-step QW operator to be ${U}_{0}={\widetilde{U}}_{1}=\mathop{R}\limits^{ \sim }{U}_{1}(\pi ){\widetilde{R}}^{\dagger }$, with $\widetilde{R}=({{\mathbb{I}}}_{c}-i{\sigma }_{y})/\sqrt{2}$, the chiral operator is *Γ* = *σ*_*x*_. The mean chiral displacement (MCD) is an observable introduced in ref. ^56^, defined as ${{\mathcal{C}}}_{x}(t):=2\langle \Gamma \widehat{x}\rangle$, with $\widehat{x}$ the lattice position operator. It can be experimentally retrieved by measuring the weighted difference between the center of mass of the intensity distributions of the two chiral projections, i.e., the projections on the eigenstates of the chiral operator, that are $| \uparrow \rangle =| H\rangle$ and $| \downarrow \rangle =| V\rangle$, where $| V\rangle$ is the vertical polarization, for the protocol considered above. When considering a walker that is initialized on a single lattice site with an arbitrary coin state, $| \psi (0)\rangle =| 0,\phi \rangle$, the MCD asymptotically converges to the winding number *ν*^56^:

17 $${{\mathcal{C}}}_{x}(t)=2\mathop{\sum }\limits_{{m}_{x}}{m}_{x}({P}_{\uparrow }({m}_{x},t)-{P}_{\downarrow }({m}_{x},t))\mathop{\to }\limits^{t\to \infty }\nu$$

**Fig. 4:**
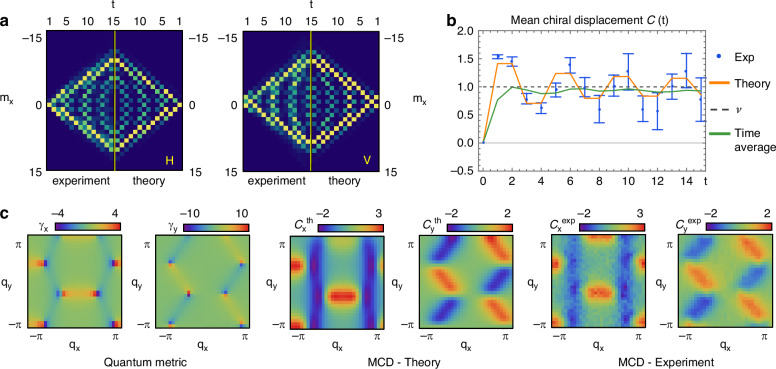
Measurement of topological features in one- and two-dimensional lattices. **a** Experimental reconstruction (left) and theoretical prediction (right) of the output distributions corresponding to the two chiral polarization components. At each time step, the probability distributions are normalized with respect to the maximum probability at that time. **b** Experimental reconstruction (blue dots) and theoretical prediction (orange line) of the MCD asymptotically converging to the winding number *ν* = 1 (gray dashed line). The green line shows the time average of the MCD. Montecarlo simulations are used to extract the error bars. Simulations are performed by assuming a maximum uncertainty of ± *π*/50 on the local birefringence of the three SLMs and averaging over 30 simulated experimental runs. **c** Quantum metric elements *γ*_*x*_ and *γ*_*y*_ (left), theoretical prediction (center) and experimental reconstruction (right) of the MCD components *C*_*x*_ and *C*_*y*_ for a 21 × 21 grid of $\vec{q}$-values in the Brillouin zone

The two chiral projections are measured through a polarization projection before the detection stage. By monitoring their evolutions (see Fig. 4a), we successfully reconstruct the MCD convergence to *ν* = 1, as expected for this QW protocol (see Fig. 4b). Our simulation refers to the evolution of a localized $| 0,H\rangle$ input across 15 time steps, thus surpassing the previous realizations of ref. ^56^ (7 steps) and ref. ^57^ (13 steps).

Note that the translation invariance of the system makes it impossible to reveal the presence of edge states, a hallmark of non-trivial topology^58–61^. The method adopted here circumvents this fundamental limitation by simply following the free evolution of a single particle, initially localized on a single site in the bulk. Measuring the MCD allows us to directly access the system topology without invoking edge effects or breaking translation invariance^56,57^.

In this experiment, error bars are estimated via Montecarlo simulations, assuming a maximum uncertainty of ± *π*/50^62^ on the local birefringence of the three SLMs, and averaging over 30 simulated experimental runs.

In our setup, the walker lattice sites are mapped into optical modes carrying (*m*_*x*_, *m*_*y*_) units of transverse momentum 2*π*/Λ. Localized states correspond to Gaussian beams with *w*_0_/*Λ* ≥ 1^44^, which are focused into separated spots in the Fourier plane. As a consequence, the walker quasi-momentum $\vec{q}$ maps into the transverse position (*x*, *y*) on the SLMs’ plane (see Methods). Within this encoding, the hologram periodicity Λ corresponds to one Brillouin Zone (BZ), with $\vec{q}\in$ BZ = [−*π*, *π*]^2^. Accordingly, preparing a narrow wavepacket peaked around ${\vec{q}}_{0}$,

18 $$| \psi (0)\rangle ={\int }_{{\mathrm{BZ}}}\frac{{{\rm{d}}}^{2}q}{{(2\pi )}^{2}}{G}_{{w}_{0},{\vec{q}}_{0}}(\vec{q})| \vec{q},{\phi }_{0}\rangle$$

where ${G}_{{w}_{0},{\vec{q}}_{0}}={\mathcal{N}}\exp (-{(\vec{q}-{\vec{q}}_{0})}^{2}/{w}_{0}^{2})$, with ${\mathcal{N}}$ a normalization factor, corresponds to preparing a beam with *w*_0_/Λ ≪ 1 in the (*x*, *y*) plane, centered in (*x*_0_, *y*_0_) = (*q*_0*x*_, *q*_0*y*_)Λ/2*π*, having polarization $| {\phi }_{0}\rangle$. Our approach allows for the preparation of these wavepackets without making any changes to the setup. Instead of preparing a smaller beam waist as done in previous works^44,63^, we expand the periodicity of the holograms displayed on the SLMs so that the beam is still covering the same region of each SLM, but this now corresponds to only a portion of the BZ. Then, we digitally shift the holograms so that the center of the beam matches ${\vec{q}}_{0}$. In so doing, our platform grants parallel access to the simulation of localized (delocalized) wavefunctions in the quasi-momentum space by simply “zooming-in” (“zooming-out”) the computed holograms. In our experiment, we specifically target the evolution operator generated by a 2D graphene-like chiral-symmetric Hamiltonian, having chiral operator *Γ* = *σ*_*z*_. The eigenstructure of this model is detailed in the Supplementary Material. In ref. ^63^, some of us showed that the MCD of wavepackets sharply peaked in the quasi-momentum space of tight-binding models featuring chiral symmetry is directly related to the elements of the quantum metric *γ*^64,65^, whose components are defined as ${\gamma }_{i}=({\bf{n}}(\vec{q})\times {\partial }_{{q}_{i}}{\bf{n}}(\vec{q}))\cdot \widehat{{\bf{z}}}$, where **n** is the pseudo-spin Bloch eigenstate^52^. The MCD components along the *x* and *y* directions for an input $| \psi (0)\rangle$ are^63^

19 $${{\mathcal{C}}}_{i}(t)=2{\int }_{BZ}\frac{{d}^{2}q}{{(2\pi )}^{2}}| {G}_{{w}_{0},{\vec{q}}_{0}}(\vec{q}){| }^{2}{\sin }^{2}(tE(\vec{q})){\gamma }_{i}(\vec{q})$$

where *i* = {*x*, *y*} and $E(\vec{q})$ is the energy band of the system. We set *t* = 1 and compute the three holograms corresponding to the evolution obtained from the graphene Hamiltonian with flat bands:

20 $${{\mathcal{U}}}_{g}(x,y)\equiv {{\mathcal{U}}}_{g}(\vec{q})={e}^{-i\bar{E}{\bf{n}}(\vec{q})\cdot \widehat{{\boldsymbol{\sigma }}}}$$

where $\widehat{{\boldsymbol{\sigma }}}=({\sigma }_{x},{\sigma }_{y},{\sigma }_{z})$ is the vector of the three Pauli matrices, and we choose $\bar{E}=\pi /2$, so that the oscillating factor ${\sin }^{2}(\bar{E}t)$ is equal to 1. This allows us to experimentally reconstruct the convolution between the Gaussian wavepacket and the quantum metric via a single MCD measurement. In particular, we magnify the BZ so that $\mathop{\Lambda }\limits^{ \sim }=7\Lambda$ (see Supplementary Material for further details). We shift the three holograms to center the beam in 21 × 21 different ${\vec{q}}_{0}$ values. Then we measure the difference between the average *m*_*x*_ and *m*_*y*_ positions of the two output chiral projections, applying the lattice size scaling $\Delta {\mathop{k}\limits^{ \sim }}_{\perp }=2\pi /\mathop{\Lambda }\limits^{ \sim }$ and comparing it with theoretical predictions (see Fig. 4c) obtained from the simulation of the complete wavepacket dynamics via Eq. (19). Interestingly, in the proximity of the Dirac points, the quantum metric diverges while the MCD collapses to zero^63^. This can be understood by observing that a Gaussian wavepacket initialized in the vicinity of a Dirac point develops a ring-shaped distribution due to the spin-orbit coupling **q** ⋅ $\hat{\sigma}$ induced by the local Hamiltonian in the low-energy limit. This solid-state effect is analogous to the optical spin-orbit coupling induced by *q*-plates^66^.

#### Single-photon experiment

All the experiments reported so far were performed with classical coherent light. The circuit performs optical manipulation preserving coherence between optical modes, which results in the correct intensity output distribution. To also assess the suitability of the circuit for quantum experiments, we simulate different single-particle dynamics by employing a heralded single-photon source. In this regime, photons are observed to arrive one by one, remaining in their coherent superposition until detection.

Temporally correlated photon pairs are generated via Spontaneous Parametric Down-Conversion (SPDC), then separated and coupled into single-mode fibers. One channel is used as a herald, while the other photon undergoes the QW. The SPDC source is well described in Methods. Both photons are subsequently directed onto an event-based camera (TPX3CAM) for coincidence measurements, as shown in Methods. Featuring a nanosecond time resolution^43^, the TPX3CAM enables the extraction of spatially resolved coincidences without the need for an external trigger. This comes at the cost of lower detection efficiency. By analyzing a few selected pixels corresponding to the herald and the QW region, we isolate simultaneously detected photons, which allows us to reconstruct the probability distribution resulting from the QW.

**Fig. 5:**
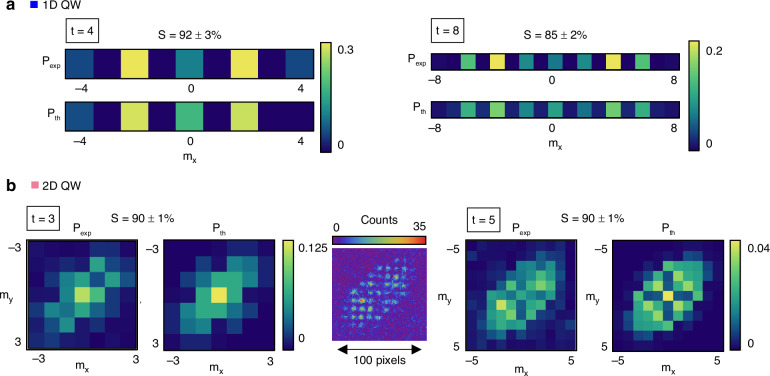
Single-photon experiment. Experimentally reconstructed (${P}_{\exp }$) and theoretically predicted ($P_{\rm{th}}$) probability distributions after **a** 4 and 8 time steps in 1D, and **b** 3 and 5 time steps in 2D QWs. Statistical uncertainties on the similarity (S) are computed assuming Poissonian statistics for photon counts. The central inset in panel (**b**) shows the output after 5 steps on the TPX3CAM (256 × 256 pixels, pixel size 55.5 μm), following a 30-minute acquisition and post-selection of coincidence events

Figure 5 shows the extracted 1D and 2D QW distributions. Panel (a) compares experimental and theoretical results for the 1D QW, while panel (b) shows the corresponding 2D distributions using the heralded single-photon source. The central inset in panel (b) displays the TPX3CAM output after post-selecting coincidence events. Errors on the similarity (S) are extracted from Poissonian photon count statistics.

The resulting single-photon distributions closely resemble those of the laser-driven QW, mainly differing in the noise level. This is expected, as our data contains a relatively high background photon count. Although time correlations help suppress this background, a residual component persists. Further background reduction can be achieved by selecting events with a time delay away from the temporal correlation peak (where only background counts are recorded) and subtracting this offset from the coincidences. This approach enhances the signal-to-noise ratio by minimizing the background noise in the distributions.

## Discussion

We have demonstrated a reconfigurable photonic circuit implementing a wide class of unitary transformations via optical manipulation at three layers only, corresponding to commercial SLMs. Its performance has been validated both with a classical laser source and single photons, implementing more than 300 different processes, eventually coupling single input modes to thousands of output ones. We reproduced several features of topological systems, going beyond the results obtained with previous platforms^44,49,56,57,63^, also exploring larger numbers of steps across 1D and 2D models. The step-by-step analysis of QW dynamics demonstrates the benefit of reconfigurability, setting this platform apart from earlier static implementations. The complexity of the explorable evolutions is only limited by the resolution of the devices. Deviations from theoretical predictions, which increase as expected with the number of modes, are mainly attributed to the reduced resolution and larger pixel size of the last SLM used in this experiment, as well as errors introduced by the SLMs’ calibration procedure.

Our proof-of-concept demonstration lays the basis for future experiments, which will benefit from a platform that is compact, programmable, and ready to use. The proposed circuit technology targets operations featuring discrete translation invariance with increasing complexity by keeping the amount of optical losses the same. In the future, the constraint of translation invariance could be relaxed by using phase masks with irrational periodicities, as typically done to simulate quasicrystals^67^, or by implementing mode-dependent operations in the far field. Combined with reconfigurability, these features will allow us to employ the same platform to explore multi-photon quantum protocols^68^.

Beyond the realization of free-space photonic circuits, which is the focus of the present work, the platform also enables programmable, arbitrary space-dependent polarization transformations. Similar functionalities can be achieved using emerging approaches such as dielectric metasurfaces, whose design, increasingly supported by advanced numerical optimization and machine learning techniques, has enabled novel structured light manipulations^69^. In contrast to typically static metasurfaces, our platform offers electronic programmability, providing a complementary and reconfigurable route to spatial polarization control.

## Materials and methods

### QWs as space-dependent polarization transformations

QWs are realized as a sequence of coin-dependent translations and coin rotations. With the definition provided in Eq. (13), these operators exhibit translational symmetry. In our encoding, the translations are implemented by LCMSs known as *g*-plates, acting as standard polarization gratings, while the coin rotations are implemented with standard waveplates^44^. *g*-plates are thin structured media whose optical action is described by a space-dependent Jones matrix:

21 $${G}_{x}(\delta )=\left(\begin{array}{cc}\cos (\delta /2) & i\sin (\delta /2){e}^{-2i\pi x/\Lambda }\\ i\sin (\delta /2){e}^{2i\pi x/\Lambda } & \cos (\delta /2)\end{array}\right)$$

If Λ = 2*π*/Δ*k*_⊥_, a *g*-plate couples neighboring modes as defined in Eq. (1). Devices coupling modes along the *y* direction have an analogous form. A quantum walk, either in 1D or 2D, is implemented by cascading multiple such devices to realize the required steps^44^. If diffraction can be neglected, the overall action of *N* devices can be modeled as a complex, space-dependent transformation:

22 $$U=\iint {\mathrm{d}}x\,{\mathrm{d}}y\,({J}_{N}\cdot {J}_{N-1}\cdot \cdots \cdot {J}_{1})\otimes | x,y\rangle \langle x,y|$$

where *J*_*i*_ represents the Jones matrix of the *i*-th plate (with the dependence on *x* and *y* omitted for brevity). Defining ${\mathcal{U}}(x,y)={J}_{N}\cdot {J}_{N-1}\cdot \cdots \cdot {J}_{1}$, one obtains the expression in Eq. (3).

### Experimental setup

**Fig. 6:**
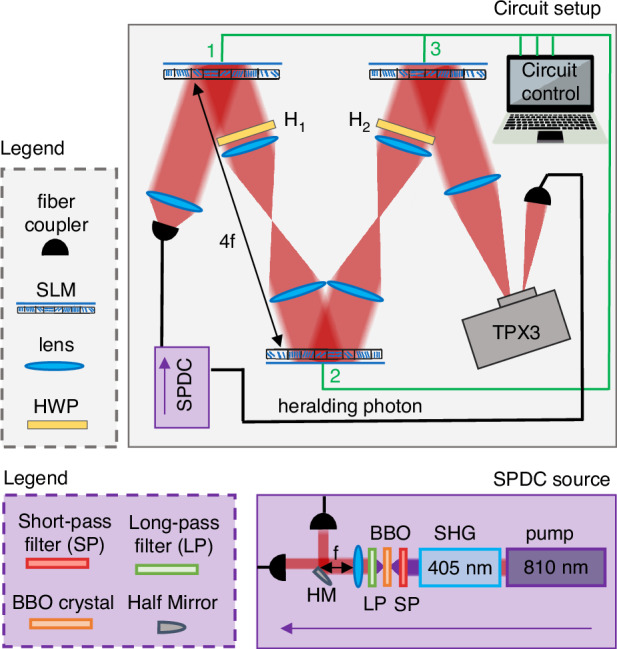
Experimental setup. The setup consists of three SLMs arranged in a relay imaging system and simultaneously controlled by a LabVIEW software (Circuit control). The second SLM is sandwiched between two HWPs, *H*_1_(22. 5°) and *H*_2_(− 22. 5°). A two-photon source based on SPDC produces pairs of temporally correlated photons, with one photon routed to the circuit and the other detected directly by the TPX3CAM camera to herald the potential arrival of its partner. The source is based on a Ti:Sa pulsed beam at 810 nm (pulse duration 120 fs, repetition rate 1 GHz, output power 2 W), which is frequency-doubled via Second Harmonic Generation (SHG) in a nonlinear crystal, yielding laser light at 405 nm with 200 mW average power. After SHG, the beam is focused (*f* = 400 mm) onto a *β*-Barium Borate (BBO) crystal cut for type-I phase matching. Idler and signal photons are separated using a half mirror (HM) placed in the far field of the crystal. Experiments with classical light are carried out by replacing the SPDC source with a spatially filtered 810 nm diode laser

A spatially filtered 810 nm diode laser source was used to obtain experimental results reported in the previous sections. A heralded single-photon source was used instead to perform a QW experiment in the single-photon regime. In this case, strongly correlated photon pairs were generated via SPDC and coupled to single-mode fibers, with one photon serving as the herald and the other undergoing the QW. The SPDC source is described in Fig. 6. A Ti:Sa pulsed beam at 810 nm (pulse duration 120 fs, repetition rate 1 GHz, output power 2 W) is frequency-doubled via Second Harmonic Generation (SHG) in a nonlinear crystal, yielding laser light at 405 nm with 200 mW average power. After SHG, the beam is focused (*f* = 400 mm) onto a *β*-Barium Borate (BBO) crystal cut for type-I phase matching. Idler and signal photons are separated using a half mirror (HM) placed in the far field of the crystal.

The setup consists of three LCOS-SLMs by Hamamatsu arranged in a relay imaging system. SLM-1 and SLM-2 belong to the X13138 series, with 1272 × 1024 pixels and a 12.5 μm pixel pitch, while SLM-3 belongs to the X10468 series and has a lower number of pixels, 792 × 600, and a wider pixel pitch of 20 μm. As shown in Fig. 6, two 4-*f* systems with equal focal-length lenses (200 mm) image SLM-2 onto SLM-1 and SLM-3 onto SLM-2, ensuring all three SLMs lie in the same plane within reasonable error. The incidence angle on each SLM is below 9°, to ensure approximately normal incidence. SLM-2 is sandwiched between two standard HWPs at ± 22. 5°. For measuring the winding number and the quantum metric, a polarization projection is also performed to filter the two chiral polarization states through a quarter-wave plate and a polarizing beam splitter (not shown in Fig. 6)

By post-selecting photons arriving simultaneously with the heralding ones at the camera, we reconstruct the distribution of the photons through the QW.

The input beam waist *w*_0_ and the spatial periodicity of the holograms Λ are set to *w*_0_ ≃ Λ = 4. mm. A crucial step in the experiment is aligning the three holograms displayed on the SLMs. The second and third SLMs are aligned with respect to the first by matching the center of their holograms to that of the first SLM in the image plane. However, this method introduces some uncertainty about the exact center positions. To refine the alignment, the classical laser source is sent through the setup, and the positions of the hologram centers are fine-tuned by optimizing the resulting QW distributions. An additional calibration step is performed to ensure that each gray level encodes the correct phase in the range [0, 2*π*] for each SLM. This method relies on the assumption that a blazed grating produces maximal efficiency in the first diffraction order and, ideally, all power would be routed into that order.

### Automatic control

We developed a control software in order to synchronize the SLMs and data acquisition using LabVIEW 2021. Each SLM acts as a monitor and is controlled over HDMI, displaying grayscale holograms with bit depth values ranging from 0 to 255, representing phase changes of 0 to 2*π*. The displayed holograms can be repositioned or replaced in real time, with a camera placed in the focal plane of the Fourier lens after the third SLM used for data acquisition. This camera’s feed is displayed live in the control panel to allow for real-time analysis and control of the experiment.

In order to calculate the similarity of the distributions for 1D and 2D QWs as described in Eq. (14), the central walker site is located and placed within a square on the camera feed. For each step in the QW, the corresponding holograms are displayed, followed by the addition of new walker spots to accommodate the increased number of steps. Each walker site is assigned a probability corresponding to the total intensity at that walker site divided by the total intensity within all walker sites. This is then the experimentally measured probability distribution. To achieve the reconstruction of the MCD, the centroid of the intensity distribution at the camera is captured and recorded for each of the 21 × 21 positions of the holograms. The *x* and *y* values of the centroid are recorded for each measurement.

### Analytical solutions for the holograms

Here, we provide the analytical solutions to Eq. (7). In our optical encoding, the mode mixing *U* can be conveniently visualized as a space-dependent polarization transformation^35,36^:

23 $$U=\iint {\mathrm{d}}x\,{\mathrm{d}}y\,\,{\mathcal{U}}(x,y)| x,y\rangle \langle x,y|$$

with ${\mathcal{U}}$ an SU(2) operator:

24 $${\mathcal{U}}(x,y)=\cos E(x,y){\sigma }_{0}-i\sin E(x,y){\bf{n}}(x,y)\cdot {\hat{\boldsymbol{\sigma}}}$$

where *E* is a real parameter and ${\bf{n}}=({n}_{1},{n}_{2},{n}_{3})$ is a unit vector. The optical sequence of three SLMs and two HWPs, ${\mathcal{S}}(x,y)={e}^{-i\frac{{\delta }_{4}}{2}}{S}_{0}({\delta }_{3}){H}_{2}{S}_{0}({\delta }_{2}){H}_{1}{S}_{0}({\delta }_{1})$, is analogously decomposed as

25 $$\begin{array}{ll}\begin{array}{lc}{\mathcal{S}}(x,y) & =\end{array} & {s}_{0}(x,y){\sigma }_{0}\\ & -i({s}_{1}(x,y){\sigma }_{1}+{s}_{2}(x,y){\sigma }_{2}+{s}_{3}(x,y){\sigma }_{3})\end{array}$$

where

26 $$\begin{array}{rcl}{s}_{0} & = & \sin \alpha \sin \beta ,\\ {s}_{1} & = & \cos \beta \sin \alpha ,\\ {s}_{2} & = & -\cos \alpha \sin \gamma ,\\ {s}_{3} & = & \cos \alpha \cos \gamma \end{array}$$

with *α* = *δ*_2_/2, $\beta =\frac{{\delta }_{1}+{\delta }_{3}}{2}$, and $\gamma =\frac{{\delta }_{1}-{\delta }_{3}}{2}$. The dependence on (*x*, *y*) is omitted for ease of notation. Imposing ${\mathcal{U}}={\mathcal{S}}$ at each transverse position yields

27 $$\begin{array}{lcl}\sin \alpha \sin \beta & = & \cos E,\\ \cos \beta \sin \alpha & = & \sin E\sin \theta \cos \phi ,\\ -\cos \alpha \sin \gamma & = & \sin E\sin \theta \sin \phi ,\\ \cos \alpha \cos \gamma & = & \sin E\cos \theta \end{array}$$

where we used the spherical parametrization of the vector **n**: ${n}_{1}=\sin \theta \cos \phi$, ${n}_{2}=\sin \theta \sin \phi$, and ${n}_{3}=\cos \theta$. Two sets of solutions are found:

28 $$\left\{\begin{array}{l}{\alpha }_{1}={\mathrm{atan}}2({\alpha }_{x},{\alpha }_{y})\\ {\beta }_{1}={\mathrm{atan}}2({\beta }_{x},{\beta }_{y})\\ {\gamma }_{1}={\mathrm{atan}}2({\gamma }_{x},{\gamma }_{y})\end{array}\right.$$

29 $$\left\{\begin{array}{l}{\alpha }_{2}={\mathrm{atan}}2({\alpha }_{x},{\alpha }_{y})\\ {\beta }_{2}={\mathrm{atan}}2({\beta }_{x},-{\beta }_{y})\\ {\gamma }_{2}={\mathrm{atan}}2(-{\gamma }_{x},-{\gamma }_{y})\end{array}\right.$$

where atan2(*x*, *y*) is the two-argument arctangent function, which distinguishes between diametrically opposite directions, and

30 $$\begin{array}{rcl}h & = & {\cos }^{2}E-{\sin }^{2}E{\sin }^{2}\theta \,{\cos }^{2}\phi ,\\ {\alpha }_{x} & = & \cos \theta \sin E/\sqrt{1-h},\\ {\alpha }_{y} & = & -\sin E\sin \theta \sin \phi /\sqrt{1-h},\\ {\beta }_{x} & = & \sqrt{1-h},\\ {\beta }_{y} & = & -\sqrt{h},\\ {\gamma }_{x} & = & -\cos \phi \sin E\sin \theta /\sqrt{h},\\ {\gamma }_{y} & = & -\cos E/\sqrt{h}\end{array}$$

From the expressions for *α*, *β*, and *γ*, the modulations for the SLMs’ holograms *δ*_1_, *δ*_2_, and *δ*_3_ can be extracted. These are defined Modulo-4*π*, while holograms are physically defined up to 2*π*. Since *S*_0_(*δ* + 2*π*) = − *S*_0_(*δ*), we use their value Modulo-2*π*, adding a minus sign when *δ*_*i*_(*x*, *y*) > 2*π*. Figure 1b shows one of the possible sets of solutions *δ*_*i*_(*x*, *y*) extracted for a 5-step 2D QW (protocol *U*_2_, input state $| H\rangle$) that could be indistinctly used in the experiment.

## Supplementary information

Supplementary Material

## Data Availability

All data are available in the main text or the supplementary materials.
