# Supplementary material for: Compact and programmable large-scale optical processor in free space
Source: Light Sci Appl. 2026 Mar 19;15:179. doi: 10.1038/s41377-026-02236-2 (PMC13002896; doi:10.1038/s41377-026-02236-2)
Supplement: Supplementary file 1 — Supplementary Material [file 41377_2026_2236_MOESM1_ESM.pdf]

Supplementary Information for: Compact and programmable large-scale optical processor in free space

Maria Gorizia Ammendola,[1,2,3] Nazanin Dehghan,[2,4] Lukas Scarfe,[2] Alessio D'Errico,[2,4]
Francesco Di Colandrea,[2,3,*] Ebrahim Karimi,[2,4,5] and Filippo Cardano[3,*]

[1] *Scuola Superiore Meridionale, Via Mezzocannone, 4, 80138 Napoli, Italy*
[2] *Nexus for Quantum Technologies, University of Ottawa, K1N 5N6, Ottawa, ON, Canada*
[3] *Dipartimento di Fisica, Università degli Studi di Napoli Federico II, Complesso Universitario di Monte Sant'Angelo, Via Cintia, 80126 Napoli, Italy*
[4] *National Research Council of Canada, 100 Sussex Drive, Ottawa ON Canada, K1A 0R6*
[5] *Institute for Quantum Studies, Chapman University, Orange, California 92866, USA*

SUPPLEMENTARY DATA

S1. TIME-RESOLVED 2D QWs

Figure S1-S5 show the results obtained for all the time steps for the 2D QW and the electric 2D QW introduced in the main text (see Fig. 2(b) and Fig. 3(c)).

S2. SUPERDIFFUSIVE TRANSITION IN A 1D QW INDUCED BY TEMPORAL DISORDER

Figure S6 shows the results obtained for the different realizations of the disordered 1D QW experiment in the superdiffusive ($\Delta = 37.5\%$) and the diffusive ($\Delta = 87.5\%$) regime. These 5 realizations have been used to compute the average value of the $\sigma^2(t)$ in the two cases, as shown in Fig. 3(b).

S3. MEASUREMENT OF THE QUANTUM METRIC IN A 2D CHIRAL LATTICE SYSTEM

The target evolution is generated from a flat-band graphene-like Hamiltonian featuring chiral symmetry, with chiral operator $\Gamma = \sigma_z$, which takes the following Bloch diagonal form:

$$\begin{aligned}\mathcal{H}_g(q_x, q_y) = &-\sigma_x[\cos((\sqrt{3}q_y - q_x)/2) \\ &+ \cos((\sqrt{3}q_y + q_x)/2) + \cos q_x]+ \\ &\sigma_y[\sin((\sqrt{3}q_y - q_x)/2)+ \\ &- \sin((\sqrt{3}q_y + q_x)/2) + \sin q_x].\end{aligned} \tag{S1}$$

Figure S7(a) shows its eigenstates structure $n_i(q_x, q_y)$, where $i \in \{x, y, z\}$. Figure S7(b) shows one of the three holograms (δ_1) used to measure the MCD, as described in the main paper. We set the size of the magnified BZ to $\tilde{\Lambda} = 7\Lambda$. The LabVIEW routine controlling the setup (described in Methods) simultaneously shifts the three holograms and centers the beam in 21×21 different $\vec{q}_0$ values, corresponding to the real positions $(x_0, y_0) = (q_{0x}, q_{0y})\tilde{\Lambda}/2\pi$ (yellow dots).

S4. CHARACTERIZATION OF LOSSES

The circuit efficiency does not decrease significantly with the number of steps, as the number of optical components stays constant, with an average recorded total reflection of $(52\pm4)\%$ from the input to the output of the QW platform. In Table S1, we report reflectance measurements to quantify the total losses for five different numbers of QW steps ($t = 2, 4, 5, 10, 20$).

* Corresponding authors: francesco.dicolandrea@unina.it, filippo.cardano2@unina.it

QW steps	Output light (% of total)
2	54.8
4	55.0
5	53.8
10	50.6
20	46.5

TABLE S1. Measured output light intensity (% of total) after three SLMs, for $t = 2$, 4, 5, 10, and 20 QW steps.

The overall optical losses depend on the characteristics of the SLMs employed, in particular on their pixel pitch and the associated filling factor. In our setup, a single reflection from SLM-1 or SLM-2 introduces losses below 5%. Therefore, three successive reflections from SLMs of this type would result in a total loss below 15% (circuit efficiency above 85%). In the specific case of the present experiment, measured losses are higher due to the inclusion of the third SLM. The same class of experiments performed with three static LCMSs featured an average transmission of $(69 \pm 1)\%$ [1].

[1] Ammendola, M. G. et al., Large-scale free-space photonic circuits in two dimensions, *Advanced Photonics* **7**, *016006 (2025)*.

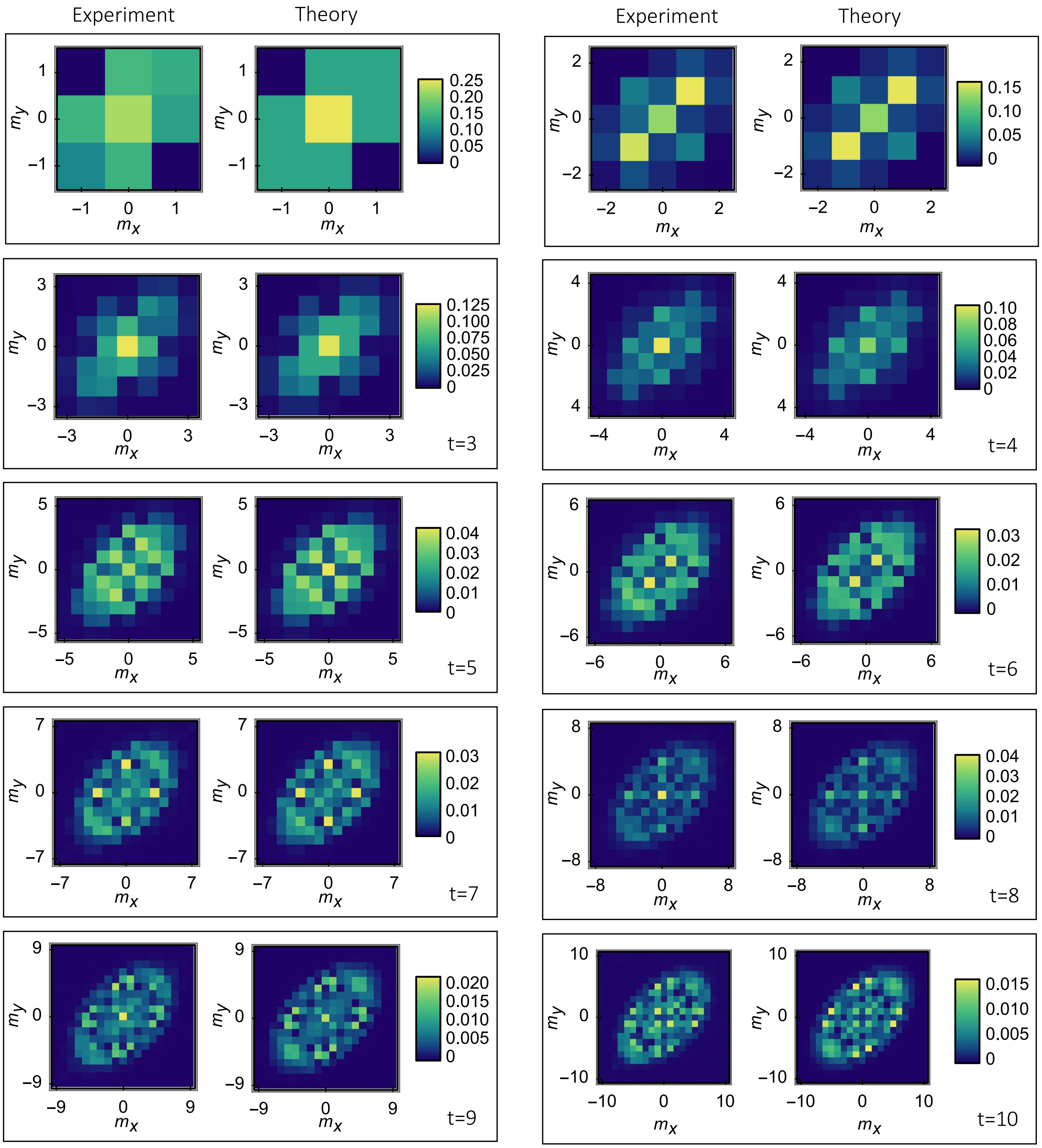

Figure S1. **2D QWs.** Experimental and theoretical distributions for each time step of the 2D QW protocol U_2, with input state $|H\rangle$, from $t=1$ to $t=10$.

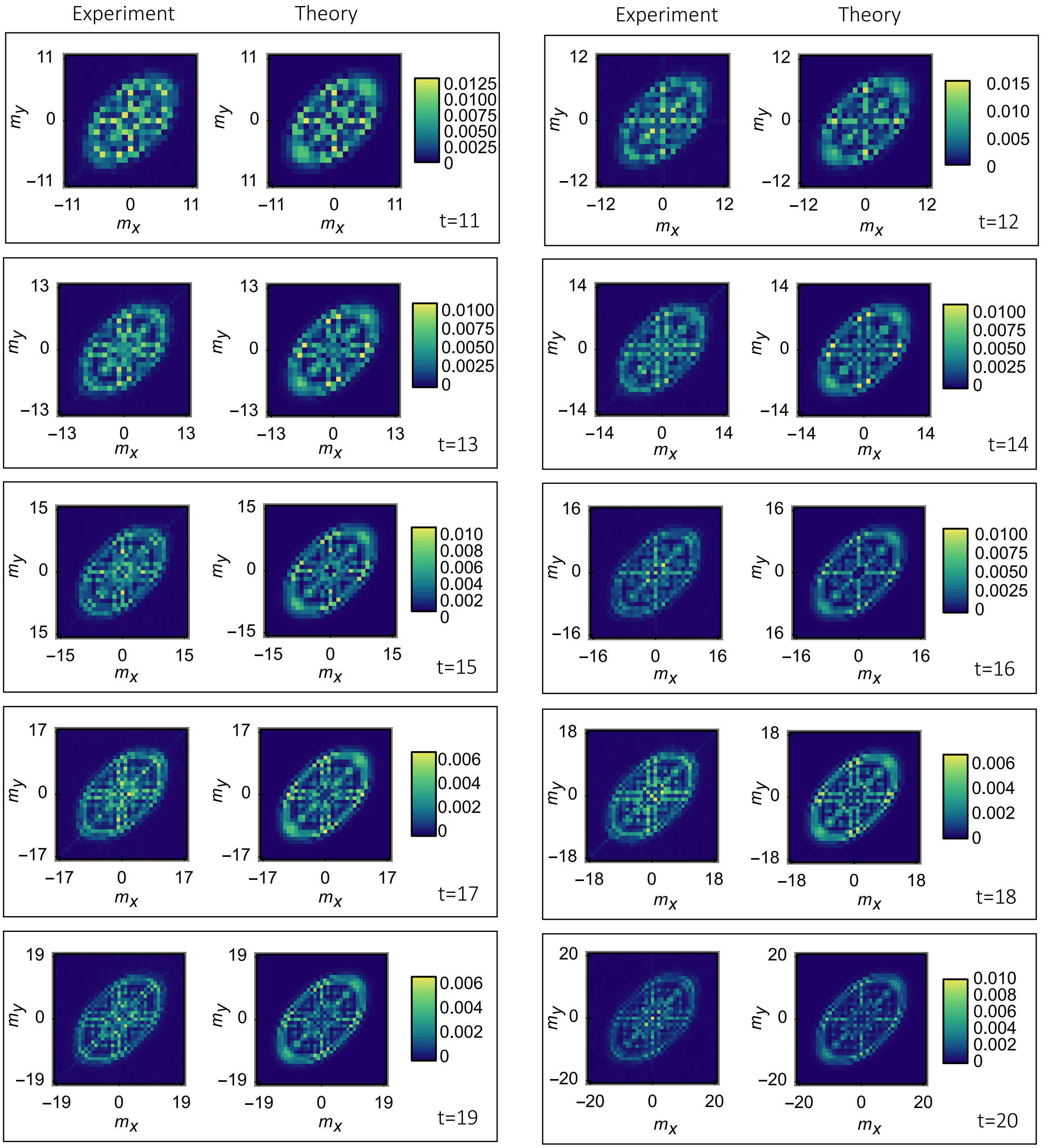

Figure S2. 2D QWs. Experimental and theoretical distributions for each time step of the 2D QW protocol U_2, with input state $|H\rangle$, from $t = 11$ to $t = 20$.

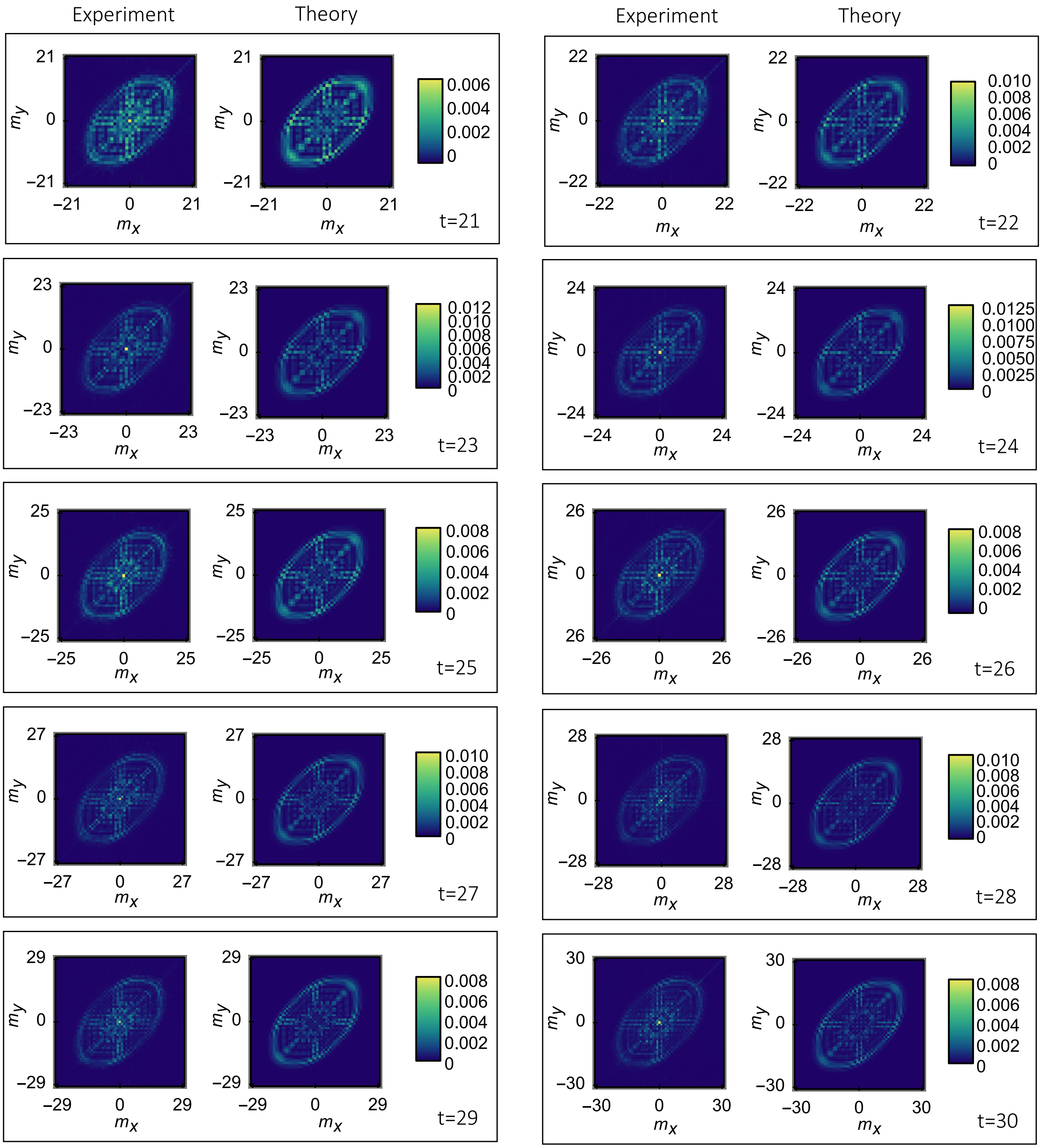

Figure S3. **2D QWs.** Experimental and theoretical distributions for each time step of the 2D QW protocol U_2, with input state $|H\rangle$, from $t = 21$ to $t = 30$.

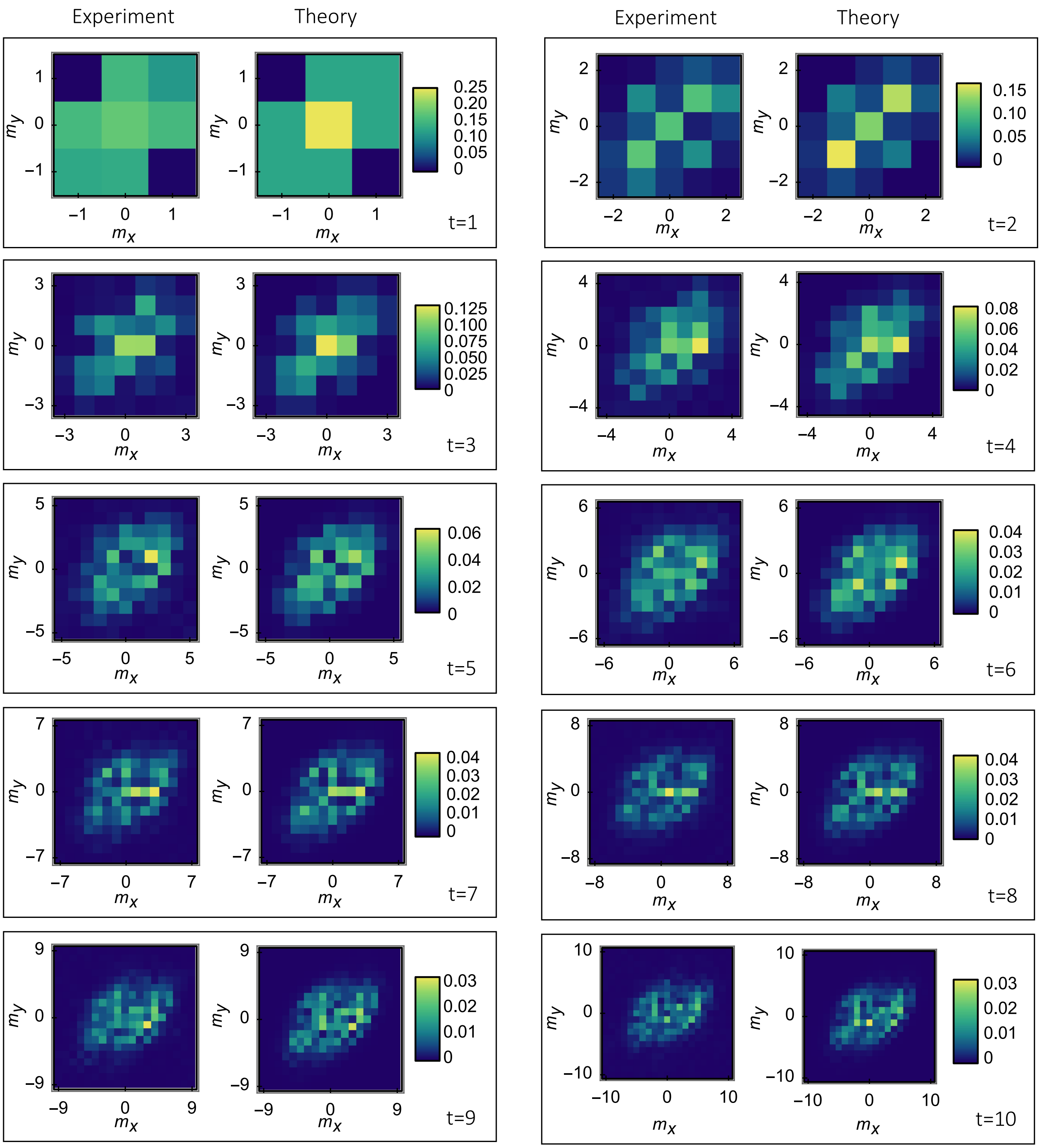

Figure S4. **Electric 2D QWs.** Experimental and theoretical distributions for a 2D QW (protocol U_2) with a constant electric field along the horizontal direction. We show each time step from $t = 1$ to $t = 10$, for an input state $|H\rangle$.

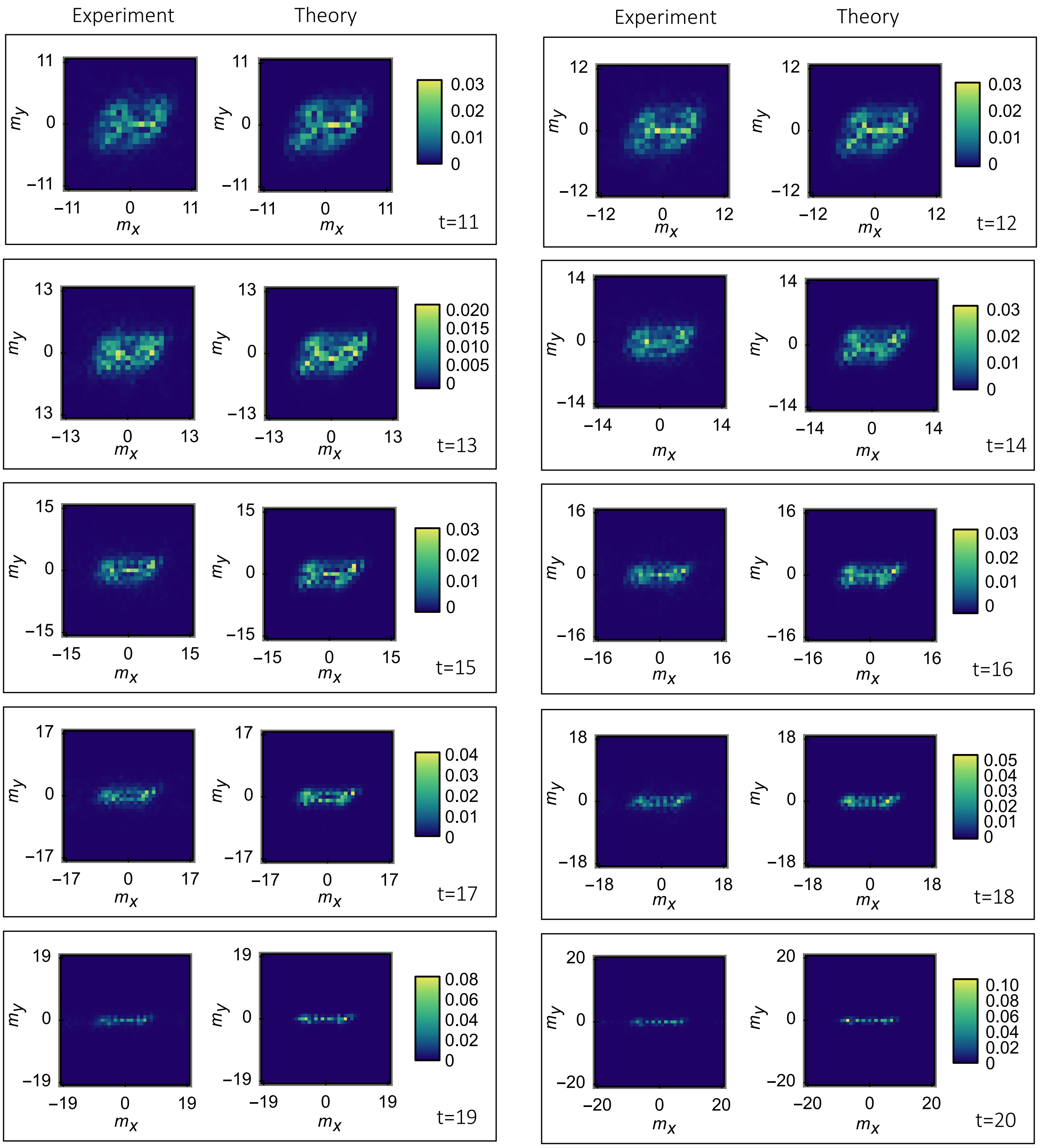

Figure S5. **Electric 2D QWs.** Experimental and theoretical distributions for a 2D QW (protocol U_2) with a constant electric field along the horizontal direction. We show each time step from $t = 11$ to $t = 20$, for an input state $|H\rangle$.

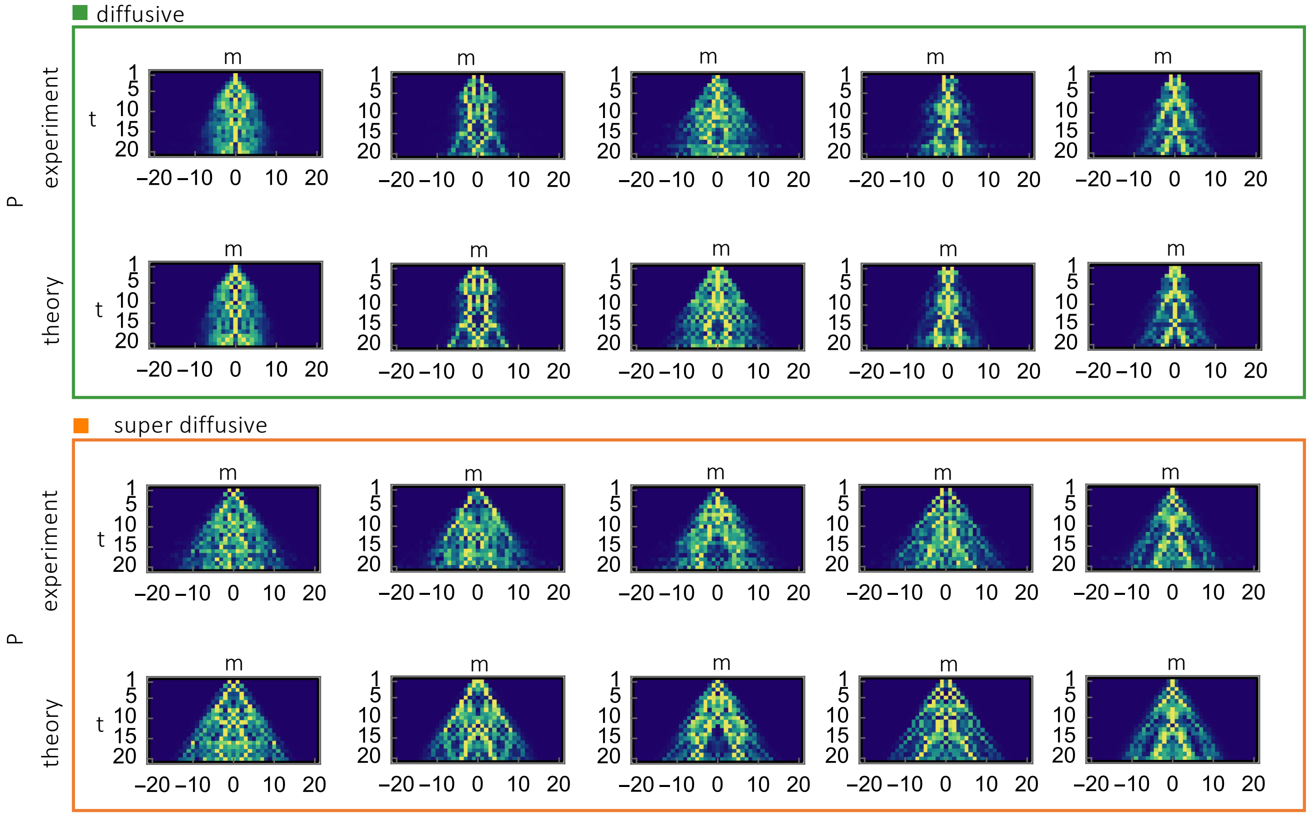

Figure S6. **Dynamical disorder in 1D QWs.** Experimental reconstruction (top) and theoretical prediction (bottom) of the output probability distribution P for 20 steps of a 1D QW with temporal disorder in five diffusive (green) and superdiffusive (orange) regimes.

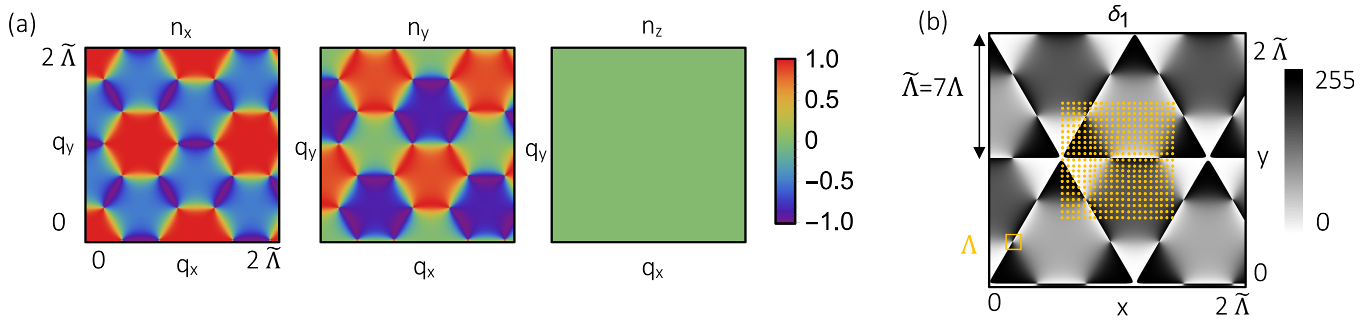

Figure S7. **Measurement of the quantum metric in a 2D chiral lattice system.** (a) Eigenstructure $n_x(q_x, q_y)$, $n_y(q_x, q_y)$, $n_z(q_x, q_y)$ of the simulated flat-band graphene-like Hamiltonian. (b) First hologram ($\delta_1(x, y)$) used for the MCD measurement. By magnifying the BZ with a 7× zoom factor, a wavefunction localized in the quasi-momentum space can be simulated. An automatic software shifts the three holograms simultaneously to simulate 21×21 different $\vec{q}_0$ values (yellow dots).
